# Platelet Rich Plasma as a Potential Therapy for Chronic Toxoplasmosis in Immunocompetent and Immunocompromised Murine Model

**DOI:** 10.3390/ph19060908

**Published:** 2026-06-08

**Authors:** Majed H. Wakid, Rabab S. Zalat, Olfat A. Hammam, Muslimah N. Alsulami, Eman S. El-Wakil

**Affiliations:** 1 Department of Medical Laboratory Sciences, Faculty of Applied Medical Sciences, King Abdulaziz University, Jeddah 22254, Saudi Arabia; 2 Special Infectious Agents Unit, King Fahd Medical Research Center, King Abdulaziz University, Jeddah 22254, Saudi Arabia; 3 Department of Parasitology, Theodor Bilharz Research Institute, Warrak El-Hadar, Imbaba, Giza 12411, Egypt; 4 Department of Pathology, Theodor Bilharz Research Institute, Kornaish El-Nile St., Giza 12411, Egypt; 5 Department of Biological Sciences, College of Science, University of Jeddah, Jeddah 21589, Saudi Arabia; mnal-sulami@uj.edu.sa

**Keywords:** *Toxoplasma gondii* ME-49, cotrimoxazole, adjunctive techniques, platelet-rich plasma, caspase-3

## Abstract

**Background**: *Toxoplasma gondii* (*T. gondii*) is one of the most prevalent parasitic zoonoses worldwide, and the host’s immunological state significantly influences its clinical manifestations, which can be potentially fatal in immunocompromised hosts. The unavailability of a vaccine, combined with the considerable toxicity of existing medications, necessitates the urgent search for new therapies or adjunctive techniques, including regenerative and immunomodulatory approaches. Hence, the present study investigated, for the first time, the therapeutic potential of syngeneic platelet rich plasma (PRP) against *T. gondii* ME49 strain-induced chronic toxoplasmosis in both immunocompetent and immunosuppressed mouse models. **Methods**: 72 albino mice were divided into two sections, immunocompetent and immunosuppressed. Each section contained six groups: healthy, model, cotrimoxazole (CTZ)-treated, PRP-treated, half-dose of both CTZ and PRP-treated, and full-dose of both CTZ and PRP-treated. Treatment efficacy was assessed via parasitological, histological, immunohistochemical, and immunological analyses. **Results**: PRP, especially when coadministered with the CTZ, mitigated the consequences of toxoplasmosis by significantly reducing brain cyst counts (*p* < 0.0001), restoring brain tissue architecture, modulating apoptotic pathways by restoring caspase-3 expression in the brain, and normalizing systemic IFN-γ, TNF-α, and IL-10 cytokine profiles. **Conclusions**: The findings highlight PRP as an adjunct to the reference treatment, CTZ, for controlling toxoplasmosis in both immunocompetent and immunosuppressed conditions via anti-infective, neuroprotective, and immunomodulatory activities.

## 1. Introduction

*Toxoplasma gondii* (*T. gondii*), an obligate intracellular protozoan, is the etiological agent of toxoplasmosis, one of the most prevalent parasitic zoonoses worldwide. Although prevalence varies significantly by region, with seroprevalence rates ranging from 10% to 80% across populations, one-third of the world’s population is estimated to be infected with *T. gondii* [1].

Almost all warm-blooded hosts, including humans, act as intermediate hosts in the parasite’s intricate life cycle, with felids serving as the definitive host. Usually, intermediate hosts become infected when they consume oocysts from tainted food or water or tissue cysts (bradyzoites) in undercooked meat. Congenital infection, blood transfusions, and organ transplants are other reported ways of transmission [2]. This variety of transmission pathways reflects the parasite’s ecological diversity and global reach.

The host’s immunological state significantly influences the clinical manifestations of toxoplasmosis [3]. In immunocompetent hosts, the primary infection is frequently asymptomatic or presents as a mild, self-limiting flu-like illness with lymphadenopathy. After the acute phase, the parasite transforms into the dormant bradyzoite form, which typically encysts in the heart tissue, skeletal muscle, and central nervous system, resulting in a latent or chronic infection that can last a lifetime. This chronic infection is concerning because it was previously considered benign. Still, it has been accused of being the etiology of neuropsychiatric disorders, such as impaired psychomotor performance, enhancement of suicide, and traffic accidents [4,5]. On the other hand, reactivating bradyzoite cysts in immunocompromised individuals, such as those with AIDS, organ transplant recipients, or cancer patients, can result in serious and potentially fatal illnesses, including *Toxoplasma* encephalitis [1].

Toxoplasmosis has a limited number of therapeutic options. For acute toxoplasmosis, the first-line treatment targeting the rapidly multiplying tachyzoite is the pyrimethamine-sulfadiazine combination, which is frequently complemented with folinic acid to reduce pyrimethamine myelotoxicity [6]. Alternatively, cotrimoxazole, which is a combination of trimethoprim and sulfamethoxazole, is also used. Significant challenges limit the first-line therapeutics, including severe adverse effects in the form of hematological toxicity, gastrointestinal discomfort, and skin reactions. Furthermore, they are ineffective against the chronic bradyzoites that form the tissue cysts, which is particularly important in immunocompromised hosts, where latent infection reactivation can result in life-threatening illness [7].

The lack of a vaccine and a tissue-cyst-eliminating treatment, combined with the considerable toxicity of existing medications, necessitates the urgent search for new therapies or adjunctive techniques, including natural products, regenerative approaches, and immunomodulatory strategies [8,9,10,11].

Platelet rich plasma (PRP) is described as an autologous product with a highly concentrated platelet suspension in a small volume of plasma with a platelet count above the baseline [12,13]. PRP’s therapeutic benefits are attributed to the alpha (α) granules found within platelets, which render PRP a significant tool in biomedicine. These α granules serve as a storage pool for several growth factors, including platelet-derived growth factor (PDGF), insulin-like growth factor (IGF), fibroblast growth factor, vascular endothelial growth factor (VEGF), platelet-derived angiogenic factor (PDAF), and transforming growth factor-beta (TGF-β) [12,14]. Moreover, platelet α-granules release bioactive proteins, such as vitronectin, fibronectin, and thrombospondin, which attract mesenchymal stem cells, osteoblasts, and macrophages [15]. Other substances are present in platelet α-granules, including catecholamines, osteonectin, serotonin, proaccelerin, and von Willebrand factor [14]. Consequently, PRP is widely utilized in regenerative medicine to enhance tissue regeneration, expedite wound healing, and reduce inflammation in various medical disciplines, including dermatology, orthopedics, ophthalmology, and dentistry. These effects are mediated by growth factors, which promote angiogenesis, cell proliferation, differentiation, and migration [13].

Besides its regenerative activities, there is growing evidence that platelets and their derivatives have both direct and indirect antimicrobial effects against a variety of infections, including parasites, through kinocidins and antimicrobial peptides that can interact with immune cells and pathogens to prevent infection. PRP has been shown to have antibacterial [16] and antifungal [14] efficacy. Moreover, PRP was used to treat parasitic infections. Antihelminthic effects of PRP were evident against *Schistosoma mansoni* [17] and *Trichinella spiralis* [18,19]. While the antiprotozoal activities of PRP have been reported against cryptosporidiosis [20], malaria [21], and leishmaniasis [22], the antitoxoplasmosis potential of PRP has been investigated previously using either heterogeneous PRP or only an immunosuppressed model [23,24]. Hence, the current study investigated, for the first time, the therapeutic potential of syngeneic platelet rich plasma (PRP) against *T. gondii* ME49 strain-induced chronic toxoplasmosis in both immunocompetent and immunosuppressed mouse models.

## 2. Results

### 2.1. Parasitological Results

The present study investigated the impact of PRP, alone or in combination with CTZ, on reducing ME49 *T. gondii* strain brain cysts in infected mice, using two treatment groups: immunocompetent and immunocompromised.

**Figure 1:**
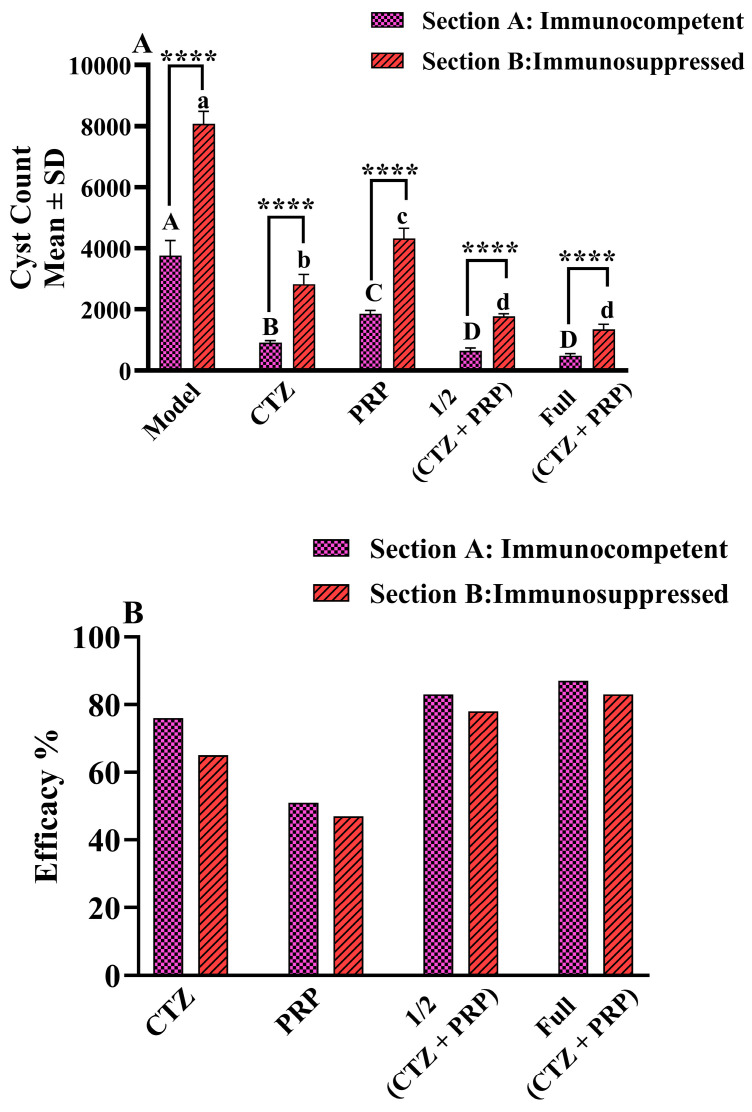
(**A**) represents *T. gondii* brain cysts number in the experimental groups of both Section A and Section B. Differences between Section A and Section B were regarded as significant at **** *p* < 0.0001. At the same time, differences between different groups in the same Section were indicated by uppercase letters for Section A and lowercase letters for Section B, where columns with letters show whether the mean values are similar (represented by the same letters) or significantly different (represented by distinct letters). (**B**) represents the inhibition percent of *T. gondii* brain cysts in both Section A and Section B.

In both sections, there was a highly significant decrease in the mean *T. gondii* brain cysts number in all treated groups (*p* < 0.001) compared with the Model (infected, untreated) group (Table 1, Figure 1). The least mean cyst count was found in GVI, which received full dose of both PRP and CTZ, with a drug efficacy of 87% and 83% in section A and section B, respectively. Notably, in both sections (A and B), there was no significant difference between the GVI and GV, which received a half dose of both PRP and CTZ (Table 1, Figure 1).

A two-way ANOVA was used to assess the concurrent effects of the two tested factors, host immunological state and treatment interventions, and exhibited a highly significant interaction between them (F(4, 50) = 82.25, *p* < 0.0001). According to the current study, the host’s immunological status (Row Factor) had a highly significant main effect, accounting for 23.34% of the total variation (F(1, 50) = 993.7, *p* < 0.0001). In the same way, the treatment regimen (Column Factor) also showed a very significant main effect, accounting for 67.76% of the total variation (F(4, 50) = 721.2, *p* < 0.0001).

Additionally, the results of Tukey’s multiple comparisons test, performed after the two-way ANOVA, indicated that the mean *T. gondii* brain cyst number in each treated group in immunocompetent mice differed significantly (*p* < 0.0001) from its matched group in immunosuppressed mice (Figure 1A).

Table 1 displays the mean ± SD of the ME49 *T. gondii* oocyst brain cyst count in immunocompetent and immunosuppressed mice. Each column’s superscript small letters show whether the mean values are similar (represented by the same superscript letters) or significantly different (represented by distinct superscript letters).

### 2.2. Histopathological Results

**Figure 2:**
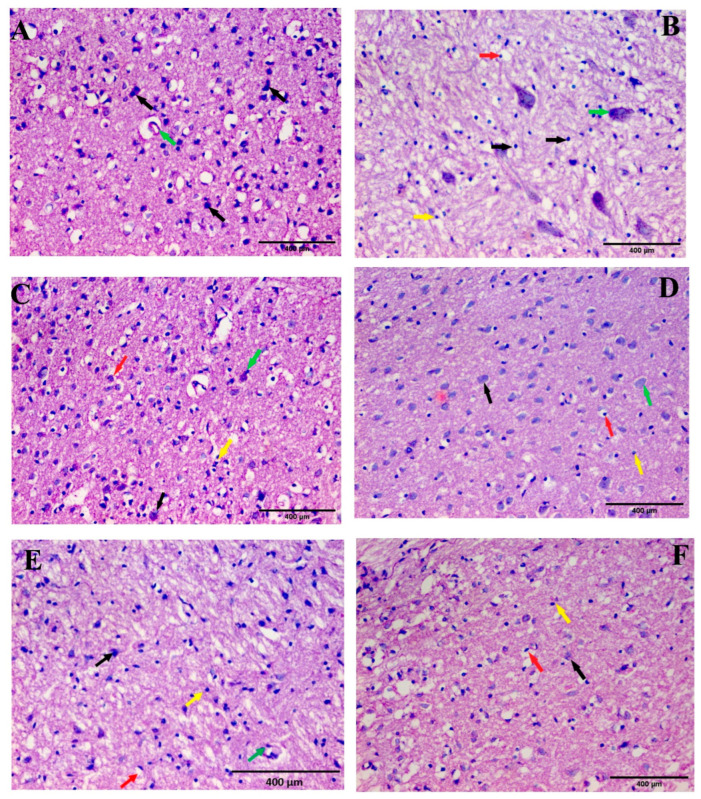
Photomicrograph of brain section from Section A, different groups: (**A**) The Control group showing many normal neuronal architectures, with central large vesicular nuclei, containing one or more nucleoli (black arrows), vascular proliferation (green arrow). (**B**) The Model group showing neuronal pyknosis and necrosis (black arrows), edema, and cellular vacuolation (red arrow), *Toxoplasma* cyst (green arrow), and inflammation as lymphocytes (yellow arrow). (**C**) The CTZ-treated group showed a moderate number of neurons with almost normal architecture, large central vesicular nuclei containing one or more nucleoli (black arrow), scattered neuronal pyknosis and necrosis (yellow arrow), edema, and cellular vacuolation (red arrow), and *Toxoplasma* cysts (green arrow). (**D**) The PRP-treated group showed many near-normal neuronal architectures with centrally located large vesicular nuclei containing one or more nucleoli (black arrow), scattered neuronal pyknosis and necrosis (yellow arrow), edema, and cellular vacuolation (red arrow), and *Toxoplasma* cysts (green arrow). (**E**) The 1/2 (CTZ + PRP)-treated group showed near-normal neuronal architectures with central large vesicular nuclei containing one or more nucleoli (black arrow), scattered neuronal pyknosis and necrosis (yellow arrow), cellular vacuolation and edema (red arrow), and vascular proliferation (green arrow). (**F**) The full (CTZ + PRP)-treated group showed a large number of near-normal neurons with central, large, vesicular nuclei containing one or more nucleoli (black arrow), scattered neuronal pyknosis and necrosis (yellow arrow), and scattered edema and cellular vacuolation (red arrow).

**Figure 3:**
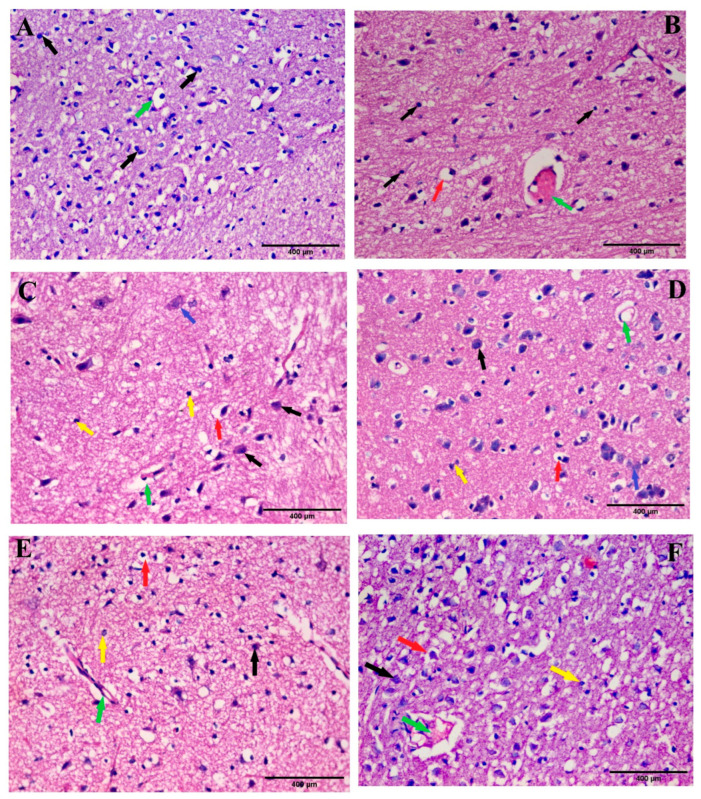
Photomicrograph of brain section from Section B, different groups: (**A**) The Control group showing many normal neuronal architectures, with central large vesicular nuclei, containing one or more nucleoli (black arrows), vascular proliferation (green arrow). (**B**) The Model group showing neuronal pyknosis and necrosis (black arrows), edema and cellular vacuolation (red arrow), and *Toxoplasma* cyst (green arrow). (**C**) The CTZ-treated group showed a moderate number of neurons with near-normal architecture with central large vesicular nuclei containing one or more nucleoli (black arrow), scattered neuronal pyknosis and necrosis (yellow arrow), edema and cellular vacuolation (red arrow), vascular proliferation (green arrow), and *Toxoplasma* cysts (blue arrow). (**D**) The PRP-treated group showed a moderate number of neurons with near-normal architecture with central large vesicular nuclei containing one or more nucleoli (black arrow), scattered neuronal pyknosis and necrosis (yellow arrow), edema and cellular vacuolation (red arrow), vascular proliferation (green arrow), and *Toxoplasma* cysts (blue arrow). (**E**) The 1/2 (CTZ + PRP)-treated group showed a moderate number of neurons with near-normal architecture with central large vesicular nuclei containing one or more nucleoli (black arrow), scattered neuronal pyknosis and necrosis (yellow arrow), edema and cellular vacuolation (red arrow), and vascular proliferation (green arrow). (**F**) The full (CTZ + PRP)-treated group showed neurons with near-normal architecture with central large vesicular nuclei containing one or more nucleoli and peripheral distribution of Nissl granules (black arrow), scattered neuronal pyknosis and necrosis (yellow arrow), and scattered edema and cellular vacuolation (red arrow) and proliferating blood vessels (green arrow).

Histopathological analysis of brain tissue sections from section A immunocompetent mice and immunosuppressed mice of section B is displayed in Figure 2 and Figure 3, respectively. Group 1, which contains the Control mice, demonstrated typical histological structures of cortical layers with apparently intact and well-organized neurons in different layers (Figure 2A and Figure 3A). On the other hand, group II, which contains the Model mice, showed diffuse records of neuronal degenerative alterations with many records of neuronal pyknosis and necrosis. Additionally, cellular vacuolation, edema, and inflammation with lymphocyte infiltration are evident, accompanied by focal records of *Toxoplasma* cysts (Figure 2B and Figure 3B).

In contrast, the CTZ, PRP, half-dose of both PRP and CTZ, and full-dose of both PRP and CTZ treatment groups showed improvement and a return to typical histological structures compared with the Model mice.

For section A, immunocompetent mice treated with CTZ (Figure 2C), PRP (Figure 3D), or half-dose PRP and CTZ (Figure 2E) showed moderate neuronal protection, with few focal areas of degenerating neurons. More abundant records of apparently intact neurons were observed, and an intact interneuronal brain matrix was shown with minimal edema, vacuolization, and sporadic glial cell infiltrates. The full-dose PRP and CTZ-treated group showed the greatest improvement, with apparently intact, well-organized neurons across layers. Furthermore, an intact intercellular matrix was observed, with minimal reactive glial cell infiltrates (Figure 2F).

Similarly, the histopathological analysis of brain tissue sections from treated mice in section B, which had immunosuppressed mice, is displayed in Figure 3C–F.

### 2.3. Immunohistochemical Results

**Figure 4:**
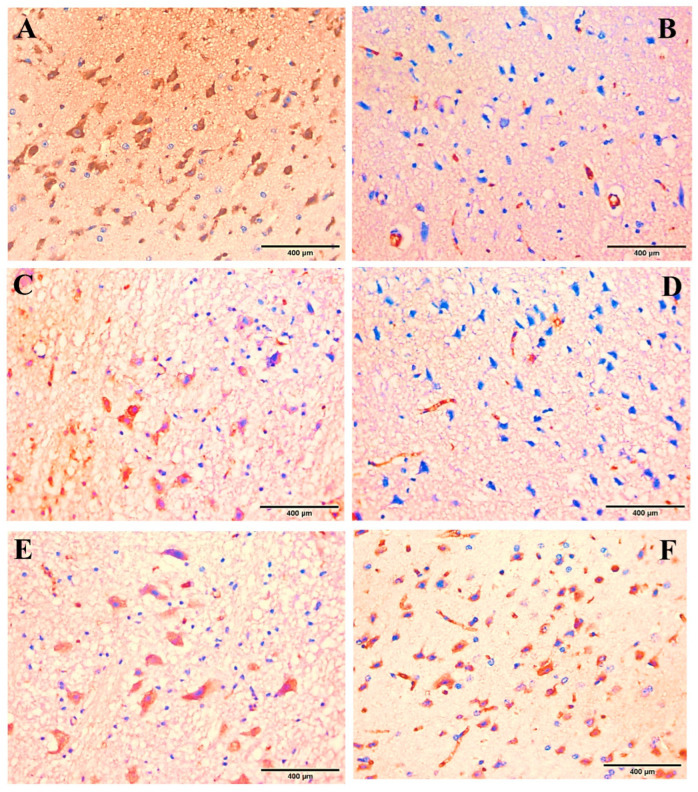
Photomicrograph of Caspase-3 IHC local expression in brain tissues from Section A, different groups: (**A**) The Control group showed a relatively high expression. (**B**) The Model group showed a very mild caspase-3 expression. (**C**–**F**) treated groups show upregulation in Caspase-3 expression.

**Figure 5:**
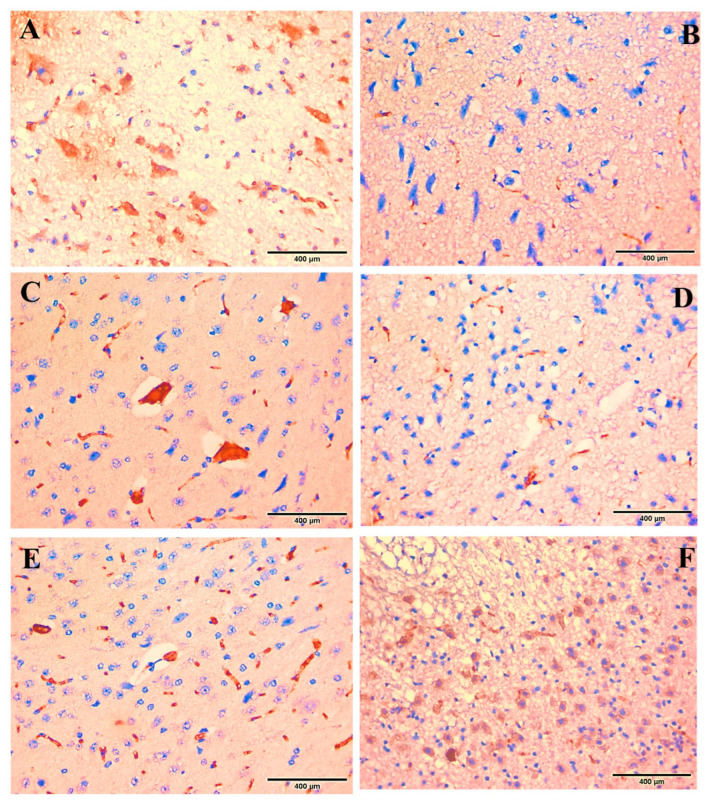
Photomicrograph of Caspase-3 IHC local expression in brain tissues from Section B, different groups: (**A**) The Control group showed a relatively high expression. (**B**) The Model group showed a very mild caspase-3 expression. (**C**–**F**) treated groups show upregulation in Caspase-3 expression.

**Figure 6:**
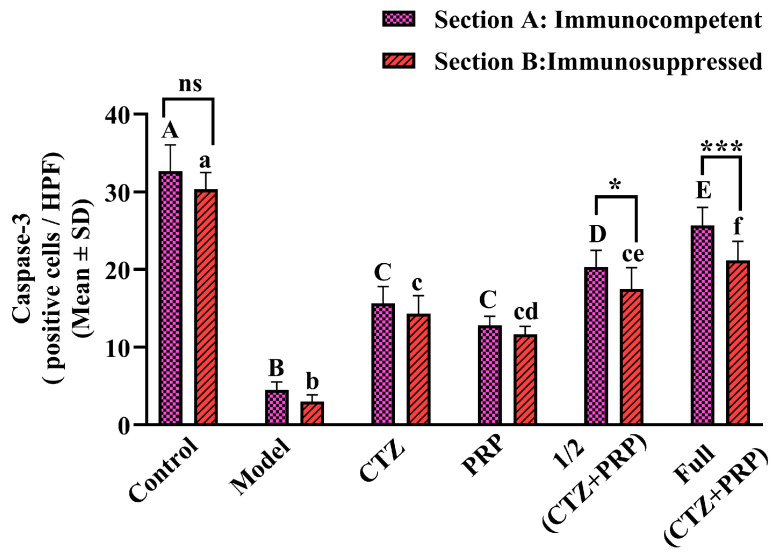
Caspase-3-positive cells/HPF in the brain tissue sections in different study groups in both Sections (A and B). Data are expressed as Mean ± SD. Differences between Section A and Section B were regarded as significant at * *p* < 0.05, *** *p* < 0.001, and non-significant (ns). At the same time, differences between different groups in the same Section were indicated by uppercase letters for Section A and lowercase letters for Section B, where columns with letters show whether the mean values are similar (represented by the same letters) or significantly different (represented by distinct letters).

Caspase-3 IHC local expression in brain tissues of the Control uninfected group in both Sections A and B exhibited strong expression (Figure 4A and Figure 5A) with no significant difference between them (Figure 6). In contrast, compared with the Control group, the cerebral cortical neurons of the Model group in both Sections A and B displayed significantly lower levels of caspase-3 (Figure 4B, Figure 5B and Figure 6).

Introduction of different treatment regimens in both Sections A and B significantly upregulated Caspase-3 expression, with the highest expression observed in the full-dose PRP- and CTZ-treated groups (Figure 4, Figure 5 and Figure 6).

### 2.4. Immunological Results

**Figure 7:**
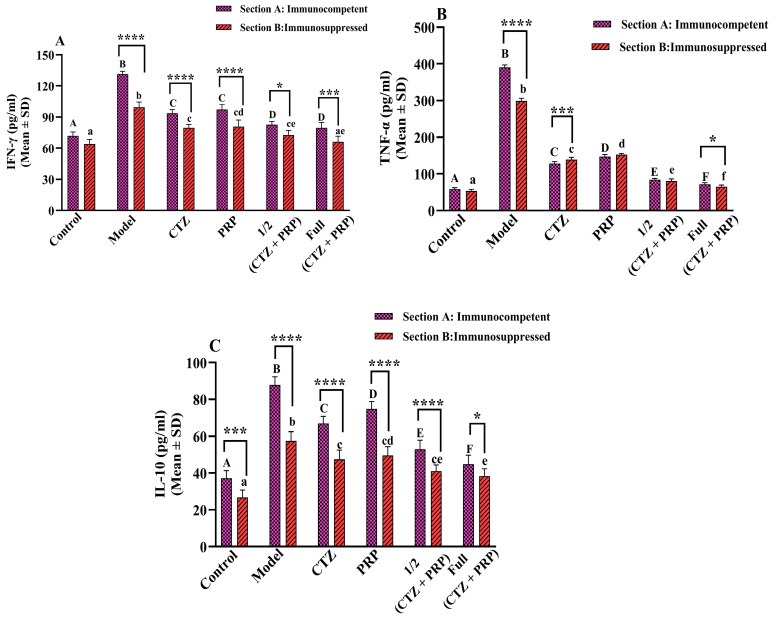
(**A**) IFN-γ, (**B**) TNF-α, and (**C**) IL-10 cytokine levels in different study groups in both Sections (A and B). Data are expressed as Mean ± SD. Differences between Section A and Section B were regarded as significant at * *p* < 0.05, *** *p* < 0.001, and **** *p* < 0.0001 or non-significant (ns). At the same time, differences between different groups in the same Section were indicated by uppercase letters for Section A and lowercase letters for Section B, where columns with letters show whether the mean values are similar (represented by the same letters) or significantly different (represented by distinct letters).

The serum levels of IFN-γ, TNF-α, and IL-10 were detected in different study groups (Figure 7A–C). Regarding the Control groups in both sections (A) and (B), the immunocompetent and immunosuppressed mice, respectively, there was no significant difference (*p* > 0.05) in IFN-γ and TNF-α levels. In contrast, the level of IL-10 was significantly lower (*p* < 0.001) in the Control group of Section B than that of Section A.

Infection with *T. gondii* induced significant upregulations (*p* < 0.0001) of all cytokines in the Model groups compared with the Control groups of both sections (A&B). Still, the cytokine levels were significantly lower (*p* < 0.001) in the Model group of Section B than in Section A.

Introduction of different treatment regimens in both sections (A and B) led to significant normalization (*p* < 0.0001) of cytokine levels compared to the Model groups, with significantly lower (*p* < 0.05) levels of IFN-γ and IL-10 in the treated groups of Section B than in Section A. At the same time, the TNF-α levels showed no significant difference (*p* > 0.05) in the PRP and the 1/2 (CTZ + PRP)-treated groups in both sections (A and B).

A two-way ANOVA was used to assess the concurrent effects of the two tested factors, host immunological state and treatment interventions, and exhibited a highly significant interaction between them in IFN-γ (F(5, 60) = 11.04, *p* < 0.0001), TNF-α (F(5, 60) = 159.6, *p* < 0.0001), and IL-10 (F(5, 60) = 13.2, *p* < 0.0001).

For IFN-γ levels, the host’s immunological status (Row Factor) had a highly significant main effect, accounting for 18.42% of the total variation (F(1, 60) = 218.8, *p* < 0.0001). In the same way, the treatment regimen (Column Factor) also showed a very significant main effect, accounting for 71.89% of the total variation (F(5, 60) = 170.8, *p* < 0.0001).

For TNF-α levels, the host’s immunological status (Row Factor) had a highly significant main effect, accounting for 0.58% of the total variation (F(1, 60) = 152.3, *p* < 0.0001). In the same way, the treatment regimen (Column Factor) also showed a very significant main effect, accounting for 96.18% of the total variation (F(5, 60) = 5086, *p* < 0.0001).

For IL-10 levels, the host’s immunological status (Row Factor) had a highly significant main effect, accounting for 25.7 3% of the total variation (F(1, 60) = 278.2, *p* < 0.0001). In the same way, the treatment regimen (Column Factor) also showed a very significant main effect, accounting for 96.18% of the total variation (F(5, 60) = 135.4, *p* < 0.0001).

## 3. Discussion

*T. gondii* is a prevalent protozoan parasite that affects populations all over the world. Given the numerous drawbacks of the medications currently used to treat *T. gondii*, including low efficacy and numerous adverse effects, there is a pressing need to develop new medications or adjunctive techniques to potentiate the etiological treatments that are both effective and minimally harmful.

In the present study, we investigated, for the first time, the therapeutic potential of syngeneic PRP as monotherapy or coadministered with CTZ against *T. gondii* ME49 strain-induced chronic toxoplasmosis in both immunocompetent and immunosuppressed mouse models. Also, the antitoxoplasmosis potential of PRP has been investigated previously using either heterogeneous PRP or only an immunosuppressed model; our study is unique in that it used syngeneic PRP and compared the two models.

*T. gondii* is one of the opportunistic protozoa that carries the risk of reactivated toxoplasmosis in immunocompromised hosts with fatal consequences. The present work aimed to simulate different health states of the human immune system, including immunocompetent and immunocompromised states, and to compare these with previously used models: either the immunocompetent model alone [23] or the immunocompromised model alone [24]. Chemical immunosuppression was induced with dexamethasone, a synthetic glucocorticoid, because its long-term use increases patients’ susceptibility to infections or the reactivation of infections [25].

PRP has been extensively investigated in regenerative medicine for its potential to treat a range of medical conditions [12]. In the current study, PRP was prepared from syngeneic mouse plasma, the same species as the recipient, to investigate its efficacy in treating toxoplasmosis compared with previously used heterologous PRP prepared from rats [24] or human plasma [23] in *T. gondii* ME 49 strain-infected mice. Although both types of PRP, either syngeneic (same species) or heterologous (different species), utilize the healing and regenerative properties of concentrated platelets, there is strong evidence that syngeneic PRP consistently performs better, primarily because of its biological advantages and lower risks [26].

In the current study, regarding *T. gondii* brain cysts count, there was a highly significant decrease in the mean number of cysts in all treated groups (*p* < 0.001) compared to the model group in both sections A and B with GVI, which received full dose of both PRP and CTZ, had the least mean cyst count with a drug efficacy of 87% and 83% in section A and section B, respectively. An enhanced effect between the antibacterial activity of CTZ, which targets the parasite’s rapidly replicating stages, and the immunomodulatory effects of PRP, which enhance host immune activity within the infected brain milieu, underlies the significant cyst clearance observed with combination therapy in our study.

These results are consistent with a previous study that found that PRP + the etiological treatment, pyrimethamine and sulfadiazine, outperformed single therapies in reducing cysts by 90% in early and 89% in late immunosuppressed *Toxoplasma* ME49 infection [24]. Our results agreed with a previous study that found that PRP + the etiological treatment, spiramycin, outperformed single therapies in reducing cysts by 83% [23]. This finding is consistent with the growing body of evidence demonstrating PRP’s therapeutic potential against parasitic diseases, including helminthic [18,27] and protozoal [20,21,22] infections.

Importantly, in both sections (A and B), there was no significant difference between the GVI, which received full doses of both PRP and CTZ, and the GV, which received half doses of both PRP and CTZ, suggesting a possible dose-sparing effect without compromising the efficacy. Still, this effect needs to be verified mathematically. Given the toxicity and poor tolerability of conventional antitoxoplasmosis regimens, lowering antibiotic dosages while preserving efficacy is of practical therapeutic importance, particularly in immunocompromised hosts. In agreement with our results, introducing PRP with β-lactam antibiotics reduced both the antibiotics’ minimum inhibitory concentrations and the colony-forming units of methicillin-resistant *Staphylococcus aureus* [28].

Regarding the histopathological examination of brain tissue sections from the Model group in both sections A and B, severe pathological alterations, including neuronal degeneration, necrosis, pyknosis, cellular vacuolation, edema, and lymphocytic infiltration, with associated *Toxoplasma* cysts, were evident. These insults could be considered a reflection of the host’s attempt to manage the persistent infection, albeit at the expense of nearby tissue damage. In agreement with these findings, several previous studies documented similar histological features in brain tissues after *Toxoplasma* ME49 infection [23,29,30,31].

The administration of different treatment regimes in both sections A and B, CTZ, PRP, coadministration of half-dose of both PRP and CTZ, and full-dose of both PRP and CTZ, mitigated the pathological alterations with the enhancement of histological architecture in these groups compared with the Model group. This enhancement may be attributed to both antiparasitic and adjuvant regeneration mechanisms. Several previous studies documented such synergistic effects of PRP when combined with other therapeutic agents in treating parasitic infections [20,23,27].

Importantly, in the immunosuppressed-treated groups in Section B, the documented improvement in degenerative alterations, coupled with the restoration of neural integrity, demonstrated that PRP’s therapeutic benefits extend beyond direct immune stimulation. This implies other processes, including the promotion of tissue healing and the regulation of the local inflammatory microenvironment.

Concerning the caspase-3 IHC local expression in brain tissues in the current study, the Control uninfected group in both Sections A and B exhibited strong expression with no significant difference between them. In contrast, compared with the Control group, the cerebral cortical neurons of the Model group in both Sections A and B showed significantly lower caspase-3 levels. While the introduction of different treatment regimens in both Sections A and B significantly upregulated Caspase-3 expression, with the highest expression observed in the full-dose PRP- and CTZ-treated groups.

Cell apoptosis has been shown to regulate the host’s response to intracellular infections caused by bacteria, viruses, and parasites. In this context, and in agreement with our findings, *T. gondii*, which regulates apoptosis, exploits caspase inhibition to persist within host cells and evade the immune system’s rapid elimination [32,33]. Additionally, previous research reported similar downregulation of caspase-3 expression in chronic experimental toxoplasmosis [30,34,35].

The upregulation observed in the Control group in the current study was in agreement with previous research [30,36]. Etewa et al. attributed this upregulation to caspase-3’s non-apoptotic roles as a regulatory protein in neurogenesis and synaptic activity [30].

Caspase-3 expression was significantly recovered after treatment, especially in group VI, which received full-dose PRP + CTZ. This upregulation points to the reactivation of apoptotic pathways in a beneficial way, which may facilitate the removal of damaged or infected neurons and parasites. In experimental models of toxoplasmosis, apoptosis was restored following therapeutic intervention, accompanied by a decrease in parasite burden [35].

Regarding immunological results, compared with the Control group, the current study showed that both immunocompetent and immunosuppressed mice with chronic *T. gondii* infection had significantly higher serum levels of IL-10, IFN-γ, and TNF-α. Immunocompetent infected animals had significantly higher cytokine levels than immunosuppressed infected mice.

The cytokine upregulation linked to persistent infection is resolved with the introduction of different therapies, as evidenced by normalization of IL-10, IFN-γ, and TNF-α levels across all treatment groups, as agreed with in previous research [37].

To prevent long-term immunopathology and promote comprehensive recovery, systemic stability is essential. While these cytokines are essential for controlling parasites, their continuous increase is detrimental, according to recent research [38,39]. This homeostatic repair is likely facilitated by PRP’s ability to trigger autophagy and modulate inflammasome activity [40].

Earlier studies by Dincel [41], focusing on nitric oxide production, oxidative stress, and glial maturation factor-beta expression in experimental toxoplasmic encephalitis, have significantly contributed to understanding the neuropathological mechanisms underlying *T. gondii* infection. More recently, the involvement of the dopaminergic system in the neuroimmunopathogenesis of toxoplasmic encephalitis has also been highlighted [42], further expanding the mechanistic framework of the disease. In this context, the current study can be considered a complementary and progressive extension of this research line, contributing to the consolidation of a coherent pathophysiological perspective and strengthening the conceptual and mechanistic foundation of the present work.

In the present study, PRP exerted its therapeutic potential, possibly via both direct antiparasitic effects via antimicrobial peptides, as evidenced by decreased brain cyst number, and immunomodulatory antitoxoplasmosis properties, as evidenced by normalization of the measured cytokines (IFN-γ, TNF-α, and IL-10).

Although there were no deaths or adverse effects associated with PRP treatment in the current study, we concur that a discussion of potential adverse effects is required for a neutral interpretation. While PRP is regarded as a safe, autologous biological product with low immunogenicity, it is nevertheless necessary to address its potential side effects. For instance, intraperitoneal PRP administration may cause temporary peritoneal irritation or a local inflammatory response. Furthermore, PRP therapy may exhibit variability in both preparation and composition, batch-to-batch inconsistencies, and potential inflammatory reactions [43,44]. Thus, although this study showed encouraging antiparasitic and immunomodulatory effects, further research on PRP standardization, optimal dosage, delivery method, and long-term safety is necessary before translation into broader therapeutic uses.

## 4. Materials and Methods

### 4.1. Ethical Approval

Under protocol number (PT: 616), the Theodor Bilharz Research Institute’s (TBRI) Research Ethics Committee (REC) approved animal studies. The National Institutes of Health’s Guide for the Care and Use of Laboratory Animals (eighth edition) is adhered to by the REC of TBRI, which functions under Federal Wide Assurance No. FWA000010609. Additionally, the ARRIVE criteria are followed in conducting the study.

### 4.2. Animals

In this study, Swiss albino male mice were obtained from the TBRI animal facility in Giza, Egypt. The mice weighed 20–30 g on average and were 6–7 weeks old. The mice were housed in air-conditioned rooms (20 ± 2 °C) in hygienic, well-ventilated plastic cages, out of direct sunlight, and with unlimited access to food and water. Moreover, before the experiment, mice exhibiting symptoms of illness, weight loss, or aberrant behavior were excluded.

### 4.3. Parasite and Infection

For parasite maintenance, Swiss albino mice were orally inoculated with brain homogenate (100 µL) containing approximately 1 × 10^2^ tissue cysts/mouse via an esophageal tube from previously infected animals provided by the TBRI’s animal house. This process was repeated every 8 to 10 weeks [45].

For infection induction, each mouse received 100 cysts/mouse at 0 week post-inoculation (wpi). At six wpi, treatments were initiated in the immunocompetent section, while in the immunosuppressed sections, treatments were initiated at eight wpi [3,45].

### 4.4. Experimental Design and Animal Grouping

G*Power 3.1.9.7 was used to perform the priori power analysis that showed a sample size of 60 was needed for a one-way ANOVA with twelve groups, with α = 0.05, power (1-β) = 0.90, and a large effect size (f = 0.70). The sample size grew to 72, with 6 mice per group, after a 20% increase to account for dropouts [46,47].

**Figure 8:**
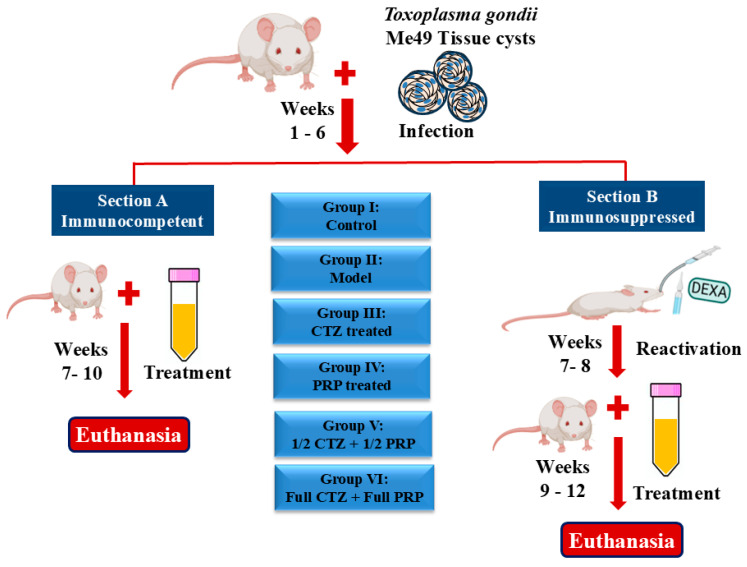
An illustration of the experimental design and animal grouping.

As shown in Figure 8, the mice were randomly divided using a computer-generated schedule into two major Sections: Section A, which was immunocompetent, and Section B, which was immunosuppressed after the establishment of infection (6–8 wpi) to induce potential reactivation [48]. Every section was further subdivided into the six groups listed below, each of which had six animals: Group I [Control]: uninfected, untreated; Group II [Model]: infected, untreated; Group III [CTZ]: infected and CTZ treated orally (370 mg/kg/day) daily for two weeks [49], (Cotrimoxazole (Septrin^®^) (Trimethoprim-sulfamethoxazole) was used as an oral suspension and provided by GlaxoSmithKline, Egypt); Group IV [PRP]: infected and PRP treated via intraperitoneal injection (0.5 mL/kg) twice per week for four weeks [20]; Group V [1/2 (CTZ + PRP)]: Infected and treated with half-dose PRP and half-dose CTZ; Group VI [Full (CTZ + PRP)]: Infected and treated with full-dose PRP and full-dose CTZ.

### 4.5. Immunosuppression

To induce immunosuppression in the animals of Section B, the immunosuppressed section, mice received oral doses of synthetic corticosteroids (dexamethasone) at a rate of 0.25 mg/g body weight daily for 2 weeks after the establishment of infection (starting from 7 wpi to 8 wpi) to induce potential reactivation. Throughout the study, dexamethasone was administered continuously [29].

### 4.6. Platelet Rich Plasma Preparation

To reduce the possibility of immunological reactivity or cross-reaction, PRP was prepared using syngeneic mouse plasma [50,51]. PRP was prepared using the double-spin technique previously described [51,52]. Briefly, 20 mice were anesthetized with light isoflurane inhalation (Forane^®^, Baxter, UK), and a capillary tube was used to draw 2 mL of blood from the mice’s retroorbital sinus, which was then placed in a sodium citrate-anticoagulant tube and centrifuged for 10 min at 400 g (1st spin). After separating the top layer as platelet-containing plasma, it was centrifuged for 10 min at 800 g (2nd spin). PRP was then extracted from the lowest third of the plasma [20]. A Medonic (DD II C) automated hematology analyzer was used to determine the platelet counts. The baseline platelet concentration ranged from 850 × 10^3^/μL to 1150 × 10^3^/μL, while the prepared PRP ranged from 3000 × 10^3^/μL to 4500 × 10^3^/μL, with a 2.6 to 5.3-fold increase relative to the baseline.

In compliance with the Guidelines of AVMA for the Animals Euthanasia [53] and the Guidelines of NIH for the Care and Use of Laboratory Animals [54], mice were euthanized under light isoflurane inhalation (Forane^®^, Baxter, UK) followed by cervical dislocation at the experiment end. The brains of the euthanized mice were harvested through a craniotomy and were individually divided into two halves. One half for counting tissue cysts in the brain, and the other for histological and immunohistochemical examination.

### 4.7. Parasitological Examination

The euthanized mice’s brain halves were individually homogenized in saline (1 mL) and sieved. The number of cysts was determined by adding 10 μL of brain homogenate to a microscope slide and counting them under light microscopy with a 40× objective. The count was performed by averaging three counts and multiplying by 100 to obtain the cyst number per mL of brain suspension (1000 μL) [45]. The mean number per brain was detected for each group.

Each drug’s efficacy was calculated according to the equation: Efficacy (%) = M–T × 100/M, where M is the mean value of the Model group, and T is the mean value of the treated group [55].

### 4.8. Histopathological Examination

The other brain halves were removed and subjected to histopathological examination to assess changes induced by *T. gondii* infection. The brain tissues were fixed in 10% formalin overnight and dehydrated in a series of graded ethanol (70%, 95%, and 100%). Then, the tissues were processed for embedding in paraffin. Histopathological sections (4 µm thickness) were stained with haematoxylin and eosin stain. After staining, the sections were examined microscopically to detect histological alterations and determine cure rates following drug administration [56].

### 4.9. Immunohistochemical (IHC) Examination

Sections were deparaffinized in xylene, mounted on slides (positively charged), and rehydrated with a series of graded ethanols (100%, 95%, 70%). The slides were heated in the appropriate phosphate buffer for 20 min to achieve antigen retrieval. A 3% H2O2/methanol solution was used to suppress endogenous peroxidase. After rinsing the sections with phosphate-buffered saline at pH 7.0, they were incubated for 24 h with a monoclonal mouse primary antibody against Caspase 3 (Santa Cruz Biotechnology, Dallas, TX, USA) at a 1:100 dilution. Lastly, immunoreactive proteins were detected by a suitable EnVision-HRP kit (Dako, Carpinteria, CA, USA), adhering to the manufacturer’s instructions. To visualize the slices, they were incubated in a 3,30-diaminobenzidine (DAB) solution (Dako, Denmark). After that, the sections were cleaned, mounted, and counterstained with Mayer’s hematoxylin. A negative control was produced for each sample and antibody. As a negative control, incubation was performed without the primary antibody. By counting every positive cell in ten high-power microscopic fields (HPF; 0.029 mm^2^ each) that identified immunopositive cells, the number of caspase-positive cells was semiquantitatively scored. The average number of positive cells per HPF was used to display the results.

### 4.10. Immunological Analysis

The serum was separated from the blood samples by centrifugation at 4500 rpm for 5 min and stored at −20 °C. Measurement of IL-10, IFN-γ, and TNF-α serum levels was performed using double-antibody sandwich ELISA assay kits from FineTest, BIOTECH Co., Ltd., Wuhan, China, according to the manufacturer’s instructions. The detection ranges for both IFN-γ and IL-10 are 15.625–1000 pg/mL. For TNF-α, the detection range is 31.25–2000 pg/mL.

### 4.11. Statistical Analysis of Data

The statistical analysis was conducted using GraphPad Prism 10.4.0 (GraphPad Software, San Diego, CA, USA). Tukey’s multiple comparisons test was performed after the two-way ANOVA test. Asterisks indicated the significance level; *p*-values less than 0.05 were deemed statistically significant. The data are expressed as mean ± SD.

## 5. Conclusions

According to the present study, coadministration of syngeneic PRP potentiates the reference treatment, CTZ, in mitigating the consequences of toxoplasmosis in both immunocompetent and immunosuppressed conditions, as documented by lower brain cyst loads, restoration of normal brain architecture, reversal of host cell apoptosis suppression caused by *Toxoplasma*, as evidenced by upregulation of caspase-3, and normalization of IFN-γ, TNF-α, and IL-10 levels. Additionally, the clinical significance of these findings extends to hosts at risk for severe toxoplasmosis, given the maintenance of efficacy in immunosuppressed mice.

## 6. Study Limitations and Future Directions

Although Type II strains (ME49) predominate in chronic murine models, future research must assess different parasite genotypes (Type I or Type III) across inbred lines (BALB/c or C57BL/6) to establish the universal translational efficacy of the tested therapeutic regimens. In the current study, reactivation of chronic toxoplasmosis was not directly confirmed by tachyzoite demonstration, peritoneal exudate analysis, or histological evidence of toxoplasmic encephalitis, despite the use of dexamethasone-induced immunosuppression to create favorable conditions for reactivation. Consequently, rather than definitively demonstrating reactivated toxoplasmosis, the results in this section should be interpreted as indicating an immunosuppressed chronic infection with potential reactivation.

Also, the current study included characterization of PRP; growth factor measurement was not performed, which is needed for reproducibility.

Despite the combination medication showing superior efficacy compared with monotherapy, mathematical synergy testing was not performed. In future studies, verifying any synergistic claims needs formal synergy testing.

Further mechanistic investigations, such as transcriptomic or proteomic analyses, are warranted to clarify PRP’s exact mode of action.

Monitoring long-term durability and relapse rates is valuable for translational outcomes.

## Figures and Tables

**Table 1:** ME49 *Toxoplasma gondii* strain brain cysts in immunocompetent (Section A) and immunosuppressed (Section B) mice.

Groups	*T. gondii* Brain Cysts			
	(Section A)		(Section B)	
	Mean ± SD	% Efficacy	Mean ± SD	% Efficacy
**Model**	3762 ± 493 ^a^		8078 ± 408.3 ^a^	
**CTZ**	910 ± 73.21 ^b^	76%	2825 ± 317.4 ^b^	65%
**PRP**	1857 ± 106 ^c^	51%	4318 ± 339.8 ^c^	47%
**1/2 (CTZ + PRP)**	642.5 ± 97.15 ^d^	83%	1773 ± 82.08 ^d^	78%
**Full (CTZ + PRP)**	490.8 ± 61.03 ^d^	87%	1348 ± 162 ^d^	83%

## Data Availability

The original contributions presented in this study are included in the article. Further inquiries can be directed to the corresponding authors.
